# Optical Tweezers in Studies of Red Blood Cells

**DOI:** 10.3390/cells9030545

**Published:** 2020-02-26

**Authors:** Ruixue Zhu, Tatiana Avsievich, Alexey Popov, Igor Meglinski

**Affiliations:** 1 Optoelectronics and Measurement Techniques Laboratory, University of Oulu, 90570 Oulu, Finland; tatiana.avsievich@oulu.fi (T.A.); alexey.popov@oulu.fi (A.P.); 2 Interdisciplinary Laboratory of Biophotonics, National Research Tomsk State University, 634050 Tomsk, Russia; 3 Institute of Engineering Physics for Biomedicine (PhysBio), National Research Nuclear University (MEPhI), 115409 Moscow, Russia; 4 Aston Institute of Materials Research, School of Engineering and Applied Science, Aston University, Birmingham B4 7ET, UK; 5 School of Life and Health Sciences, Aston University, Birmingham B4 7ET, UK

**Keywords:** optical tweezers, red blood cells, erythropoiesis, microrheology, cellular biomechanics, pathology

## Abstract

Optical tweezers (OTs) are innovative instruments utilized for the manipulation of microscopic biological objects of interest. Rapid improvements in precision and degree of freedom of multichannel and multifunctional OTs have ushered in a new era of studies in basic physical and chemical properties of living tissues and unknown biomechanics in biological processes. Nowadays, OTs are used extensively for studying living cells and have initiated far-reaching influence in various fundamental studies in life sciences. There is also a high potential for using OTs in haemorheology, investigations of blood microcirculation and the mutual interplay of blood cells. In fact, in spite of their great promise in the application of OTs-based approaches for the study of blood, cell formation and maturation in erythropoiesis have not been fully explored. In this review, the background of OTs, their state-of-the-art applications in exploring single-cell level characteristics and bio-rheological properties of mature red blood cells (RBCs) as well as the OTs-assisted studies on erythropoiesis are summarized and presented. The advance developments and future perspectives of the OTs’ application in haemorheology both for fundamental and practical in-depth studies of RBCs formation, functional diagnostics and therapeutic needs are highlighted.

## 1. Introduction

The fact that light can exert forces has been known since Johannes Kepler’s (1619) observation of a comet’s tail that always points away from the sun, and the exploration of optical forces has been revolutionized since the demonstration of laser-induced particle guiding in 1970 [1]. After nearly 50 years of comprehensive research and steady development, laser-based gradient-force optical traps, also known as “optical tweezers“ (OTs), have rapidly grown from a novelty to a widely used and indispensable tool in many fields, including single-molecule biophysics, cell biology, microchemistry, statistical physics and laser cooling and trapping of atoms [2,3,4]. Conventional and novel OTs are capable of providing small confinement geometry with a large degree of operation freedom as well as accurate detection of forces at piconewton ($10^{-12}$N) level. Single particles of a wide variety of shapes and sizes, including atoms as small as hundreds of angstroms (Å), molecules, submicron particles and macroscopic dielectric particles of hundreds of micrometers (μm) in size can be optically trapped [5].

Looking through the history of exploring the light radiation pressure, the observation of acceleration of micro-sized particles by visible laser light [1] and the successful optical trapping of dielectric particles in the Rayleigh size regime in the late 20th century [6] have laid the foundation of early optical confinement. With the first report of damage-free optical trapping of virus and bacteria [7], “optical tweezers“ (OTs) stepped onto the stage of studying living organisms and initiated far-reaching influence in analytical studies in biological sciences. OTs are not only able to safely hold onto cells and single-cell organisms, they are also capable of reaching into a large biological object and manipulating a substructure inside it [8,9]. During the first 20 years of exploration, numerous applications of OTs in biological studies, including measuring the bio-forces generated by mechanoenzymes [10], cell immobilization in cell fusion with pulsed UV laser [11] and DNA stretching for studying their mechanical flexibility [12] have begun to spring up. Direct observation of the characteristic steps of biological motors (e.g., kinesin, myosin and dynein) on a molecular scale with high spatial and temporal sensitivity has been made possible with optical trapping interferometer [13], which opened the exploration of the ingenious combination of OTs with other light-based techniques. Possible defects of OTs, i.e., the heating effects and photo-damage to biological objects, have been studied in detail [14,15,16] and are discussed in this review. Achievements during this period are properly summarized in review articles [17,18,19]. After entering the 21st century, multichannel and multifunctional OTs with great operation precision and degree of freedom have been developed and applied to a wide range of areas, including biology, physics, chemistry, colloid and aerosol sciences [2,3,20,21]. Based on specific intrinsic optical characteristics of different cells in response to an external light field, OTs offer extremely high efficiency in cell sorting [22,23]. Laser sources with frequencies far from any resonance of the trapped particles are commonly utilized in OTs, whereas wavelengths that are red-detuned from a particle’s resonant absorption peak can enhance the trapping strength and provide specificity in optical trapping [24]. Invaluable microrheological information for a better understanding of basic physics of fluids, cell membrane biophysics (e.g., surface tension and bending modulus) and molecular mechanisms underlying force-dependent biological processes have been revealed by OTs [25,26,27]. Clinical applications to disease diagnosis and treatment, e.g., studying the basic aspects of heart infarction by OTs combined with laser scissors (microbeams), have been reported [28]. Remarkably, a recent experimental study has demonstrated recognition and selective manipulation of different structures (e.g., microinjected nanoparticles, bacteria and different type of cells) in living zebrafish and analyzed the interaction detail between nanoparticles and endothelium in a cell-free in vivo environment “cleaned“ by OTs [29]. Nowadays, the breathtaking prospects of the revolutionary OTs in living cell manipulation, single-molecule biophysical analysis, cell mechanical characterization and quantitative biomechanics evaluation in sophisticated biological processes have found abundant applications in studying molecular motors, RNA folding/unfolding, intercellular communications, etc. [27,30,31,32]. The innumerable applications of OTs in the last two decades have been thoroughly reviewed with respect to cell biology and living systems [26,27,33,34], microrheology and biorheology studies [25,35], bio-forces and force-dependent biological processes [2,4,36,37], nanotechnology [3,38] and noncontact particle assembly or nanofabrication [39,40]. The great contribution of optical trapping and manipulation in biological sciences has been gloriously recognized in 2018 with half of the Nobel Prize in Physics awarded to its pioneer Arthur Ashkin.

Application of OTs is also promising in the field of blood rheology. Particularly, in recent years, the vital regulatory role of biological forces in embryogenesis, especially in the development of the cardiovascular system, has been gradually recognized and valued [41]. However, the potential role of biomechanical forces applied to the blood vessel wall in determining the hematopoietic potential at the developmental stage and the initiation and continuation of red blood cell (RBC) production are not fully understood. In this paper, the basic working principles of OTs in aspects of physical interpretation, implementation, trapping force calibration and potential thermal or nonthermal influence on the trapped biological objects are thoroughly discussed. The existing utilization of OTs-based methods in studying the erythropoiesis is presented and the emphasis is placed on the applications of OTs in direct RBC property investigations, including the mechanical and electrical properties of the RBC membrane, the cell-cell interaction dynamics and the cellular responses of RBCs in physiological and pathological conditions. Future trends of OTs in RBC-related studies, including the influence of altered cell rheology and motility on health conditions for personalized early diagnosis and a better understanding of the erythropoiesis, as well as the potential in vivo applications for therapeutic purposes are prospected.

## 2. Working Principles

### 2.1. Physical Interpretation of Optical Trapping

**Figure 1:**
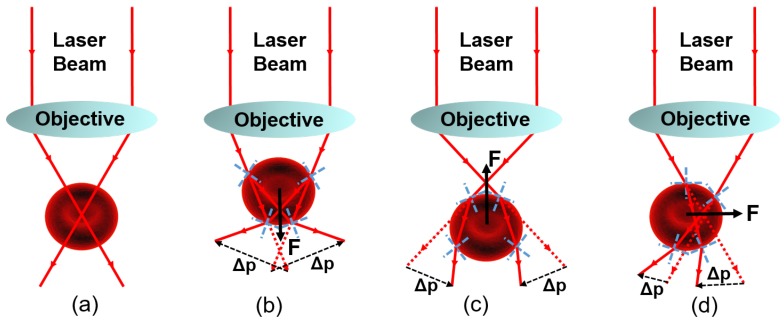
Illustration of trapping light propagation and the optical force exerted on a particle (RBC—red blood cells) that can be considered as a weak positive lens. (**a**) No momentum change as light propagates through the RBC placed at the beam focus. (**b**) Divergent refraction accompanied by a momentum increase of the RBC and the corresponding optical pushing force towards beam propagation. (**c**) Convergent refraction induces a momentum decrease of the RBC and optical force acts to pull the RBC in the opposite direction of beam propagation. (**d**) Left deflective refraction by the RBC on the left side of the beam focus results in a rightwards optical force exerted on the RBC [42].

Optical forces utilize the radiation pressure arising from the momentum of the light itself. Upon transmitting through a dielectric particle, the photons from the laser beam undergo a series of activities including absorption, scattering, emission and re-radiation, thus inducing momentum changes in both the photons and the particle interacting with them. Therefore, an optical force that equals the time rate of momentum changes is exerted on the particle [42]. The radiation force is generally divided into two components, the scattering force that is proportional to the incident light and the gradient force that is proportional to the intensity gradient. Under the gradient force, particles with a higher refractive index than the surrounding medium will be grabbed into the high-intensity region of the beam near the focus, whereas particles with a low refractive index will be pulled out toward the low-intensity area [43]. For dielectric particles in the geometric optics regime, where the particle size (diameter *d*) is large compared with the trapping wavelength (d>λ), the behavior of the laser beam can be described as a bunch of light rays [44]. The trapping phenomenon can be qualitatively understood by a simple ray-optics model that illustrates the longitudinal and transverse momentum changes arising from light-particle interaction (i.e., refraction) as shown in Figure 1. In this model, the trapped particle (RBC) with a higher refractive index than the surrounding medium is considered as a weak positive lens [42]. The resulting optical force attracts the adjacent particle towards the focal point of the laser beam. At the same time, due to light reflected by the particle, there is always a scattering force acting along the direction of light propagation. Therefore, the equilibrium position of a particle in an optical trap is slightly below the beam focus.

When the diameter of the trapped particle falls in the Rayleigh regime (d≪λ), light can no longer be represented as rays but as an inhomogeneous electromagnetic field, and the particle is then treated as a simple point dipole. It is necessary to solve Maxwell equations with appropriate boundary conditions of the given situation to calculate accurately the forces acting on the trapping particle [24]. A simple analytical result can be deduced under the approximation that the electric field is uniformly distributed across a spherical Rayleigh particle, and the optically induced dipole moment in the Rayleigh sphere can be given by [42]:

(1) $$\alpha =4\pi \varepsilon_{med}r^{3}\left(\frac{m^{2}-1}{m^{2}+2}\right)E\left(r,t\right)=4\pi n_{med}^{2}\varepsilon_{0}r^{3}\left(\frac{m^{2}-1}{m^{2}+2}\right)E\left(r,t\right),$$

where $n_{med}$ is the refractive index of the trapping medium, $\varepsilon_{med}$ is the electric permittivity of the medium, *r* is the radius of the particle (diameter: *d*) and *m* is the relative refractive index of the particle determined by the ratio of the refractive index of the particle ($n_{p}$) to the refractive index of the medium ($m=\frac{n_{p}}{n_{med}}$). The gradient force $F_{grad}$ can be written as [45]:

(2) $$F_{grad}=\frac{1}{2}\alpha \nabla E^{2}\left(r,t\right)=\pi n_{med}^{2}\varepsilon_{0}r^{3}\left(\frac{m^{2}-1}{m^{2}+2}\right)\nabla {\left|E\left(r\right)\right|}^{2}=\frac{2\pi n_{med}r^{3}}{c}\left(\frac{m^{2}-1}{m^{2}+2}\right)\nabla I\left(r\right),$$

where $I_{0}$ is the incident light intensity and the relations $E^{2}\left(r,t\right)=\frac{1}{2}{\left|E\left(r\right)\right|}^{2}$ and $I\left(r\right)=\hat{z}\frac{n_{med}\varepsilon_{0}c}{2}{\left|E\left(r\right)\right|}^{2}$ are applied in calculation. At the same time, the Rayleigh scattering force is given by [43]:

(3) $$F_{scat}=\frac{P_{scat}}{c}=\frac{n_{med}\;\cdot \;I_{0}}{c}\;\cdot \;\sigma_{s},$$

where $P_{scat}$ is the Rayleigh scattered power and $\sigma_{s}$ is the Rayleigh scattering cross-section given by [46]:

(4) $$\sigma_{s}=\frac{2\;\cdot \;\pi^{5}}{3}\;\cdot \;\frac{d^{6}}{\lambda^{4}}\;\cdot \;{\left(\frac{m^{2}-1}{m^{2}+2}\right)}^{2}.$$

Thus, the Rayleigh scattering force can be written as:

(5) $$F_{scat}=\frac{n_{med}\;\cdot \;I_{0}}{c}\;\cdot \;\frac{128\pi^{5}r^{6}}{3\lambda^{4}}\;\cdot \;{\left(\frac{m^{2}-1}{m^{2}+2}\right)}^{2}.$$

**Figure 2:**
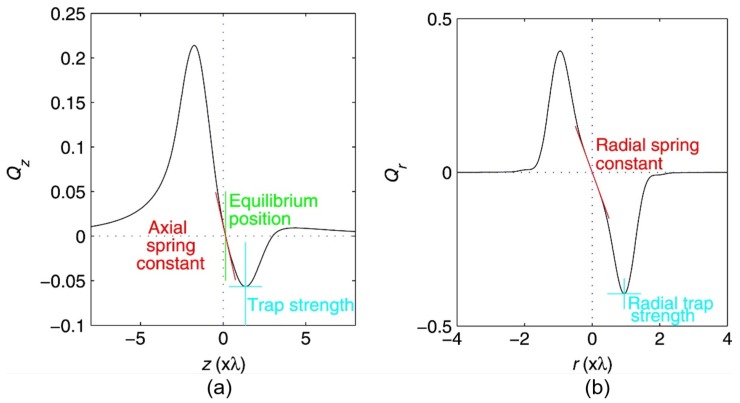
The relationships between the (**a**) axial and (**b**) radial optical forces exerted on a Rayleigh particle and their relative displacement to the equilibrium position in an optical trap [47]. The asymmetry of the axial force (in *z* direction) is due to the scattering force towards the direction of beam propagation.

The dimensionless factor *Q* indicating the trapping efficiency can be derived from the trapping force $F_{trap}$ and the incident beam power *P* by:

(6) $$Q=\frac{c\;\cdot \;F_{trap}}{n_{med}\;\cdot \;P}.$$

where *P* is the incident beam power. A typical force-displacement relationship in an optical trap is illustrated in Figure 2 [47]. Detailed physical models for quantitative and qualitative description of optical forces both in geometric optics regime and under electromagnetic theory have been well established and can be found in literature [6,42,44,45,46,47,48]. Concerning the trapping of biological objects, the optical forces exerted on optically active particles have been analytically modeled with T-matrix formalism [49]. The accurate and efficient theoretical models and calculations of optical forces are of great scientific and practical importance in understanding trapping behavior, designing trapping geometries and interpreting experimental observations.

### 2.2. Implementation

**Figure 3:**
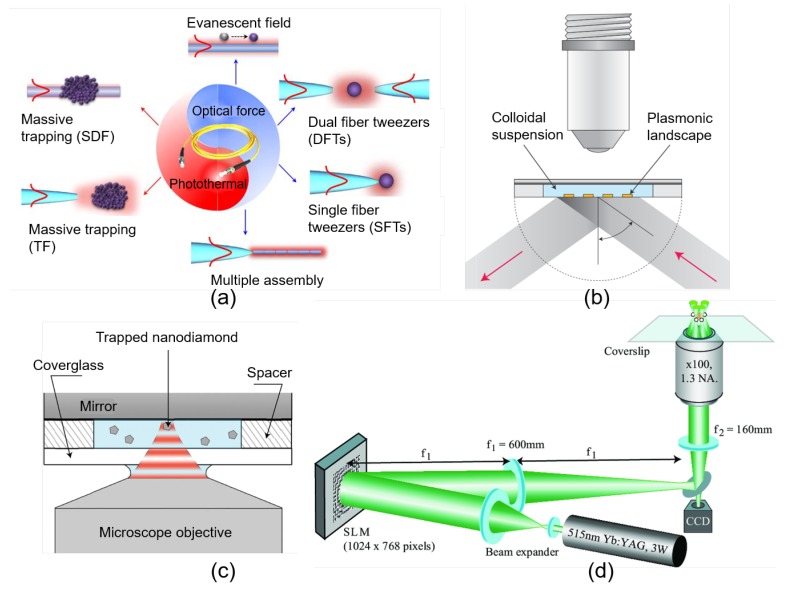
Illustration of implementation of several advanced optical trapping and manipulation techniques. (**a**) Overall elucidation of sub-wavelength diameter optical fiber (SDF) and tapered optical fiber (TF) based optical trapping established on the effects of photothermal or optical gradient field [74]. (**b**) Schematic diagram of surface plasmon (SP)-based near-field trapping realized by light source excitation of plasmonic resonance on the surface of a nanostructured substrate with required optical design [53]. (**c**) Illustration of a standing wave optical trap (SWOT) system generated by the interference between the incident and reflected laser beams within the sample chamber [54]. (**d**) Schematic diagram of a quadruple-channel holographic optical tweezers (HOTs) system. The trapping beam is reflected and reshaped into a designed pattern by the spatial light modulator (SLM) placed in the Fourier plane of the sample, which imprints the computer-generated holograms onto the wavefront [55].

In standard OTs, to achieve efficient noncontacting optical trapping and manipulation, sufficient light intensity gradient is created by tightly focusing a laser beam to a diffraction-limited spot size through a high numerical aperture (NA) objective. The simplest trapping geometry is the objective-based single-beam trap. Nowadays, multiple-trapping can be easily realized by splitting the trapping beam based on polarization [50], by time-sharing techniques (e.g., swiftly shifting one laser beam among several locations) or by trapping-beam shaping techniques (e.g., using diffractive optical elements) [51]. Several advanced optical trapping techniques including fiber tweezers [52], plasmonic OTs [53], standing wave optical trap (SWOT) [54] and holographic optical tweezers (HOTs) [55] are illustrated in Figure 3. The SWOT capable of producing deep potential wells for efficient free-nanoparticle trapping and transporting in solution is one of the typical interferometric OTs, in which the optical gradient field is created by the light interference fringes [56,57,58]. The near-field two-dimensional (2D) OTs with controlled surface plasmonic fields bound to a metal-dielectric interface can provide parallel and selective trapping of dielectric beads through nonfocused illumination with significantly reduced laser energy density compared with traditional optical trapping [53,59,60]. The use of spatial light modulator (SLM) further simplifies the generation of the complicated spatial distribution of the trapping light field and enhances the functional capabilities of the OTs systems [61]. Computer-generated HOTs with arbitrarily distributed trapping arrays make it possible for creating well-designed multiple traps and are extraordinarily beneficial to the nanofabrication of three-dimensional complex structures [62,63,64]. Remarkably, conventional far-field OTs can apply sufficient trapping forces upon micron-scale particles within diffraction limit, whereas the advanced near-field OTs can overcome the diffraction limitation and optically confine nanoscale particles in the Rayleigh regime [60]. In general, the development of novel multichannel and multifunctional OTs has provided great capability in optical fractionation [62], laser guiding (transport) of particles along defined pathways [65] and nanotechnology [58].

**Figure 4:**
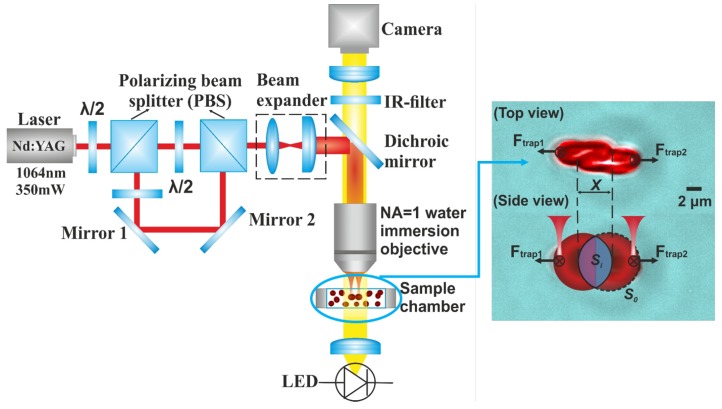
Schematic diagram of a typical two-channel optical tweezers system used for RBCs studies [73]. Two trapping channels are formed by focusing two light beams divided by polarizing states from a single laser source through a large numerical aperture objective. Insert top view image demonstrates two trapped RBCs in contact with each other with a linear overlapping distance of *x* and a corresponding contact area of $S_{i}$. The total surface area of a single RBC is denoted by $S_{0}$.

Particularly, spin and orbital angular momentum of the trapping light can be transferred to the trapping particles using modern tools including birefringent optical component, spiral phase plates, spatial light modulators, or q-plates [66]. For instance, weakly absorbing objects were rotated and moved along a given trajectory under the trapping beams with designed intensity and angular momentum distributions generated with special phase and amplitude-phase masks [67]. Advanced optical trapping with rotational manipulation has made it possible to introduce twist or apply torque to a biological system as well as to detect and analytically study the rotational movement of biological objects (e.g., bacterial flagellar motor) [32,68,69]. Attention has also been placed on building inexpensive OTs with commercially available optics and mounts for educational purposes [70,71]. Detailed guidance on the construction of different optical manipulation systems, including conventional single-beam OTs based on a homemade microscope, advanced holographic OTs and speckle OTs based on multi-mode optical fiber can be found in literature [72]. In studies of the characteristics of blood components and rheology at a single-cell level, the easy-to-implement and well-developed conventional dual-beam OTs are the most commonly used trapping geometry. In a typical dual-beam OTs system as shown in Figure 4 [73], by using polarizing beam splitters (PBS), one infrared laser beam is split into two polarization-based beams and focused by a large numerical aperture microscope objective. With beam positions being adjustable by rotating reflective mirrors or by moving the sample stage, two individual RBCs can be trapped and controlled simultaneously. Top-view monitoring and recording of the sample chamber enable further calculation and analysis of experimental observations.

### 2.3. Trapping Force Calibration

The trapping strength in OTs is directly determined by laser intensity and is dependent on the laser wavelength and the size, shape and optical properties of the trapped object [75]. In particular, the influence of the particle shape on the magnitude and orientation of optical forces is more dominant in the geometric optics regime (d>λ) than in the Rayleigh regime (d≪λ) [7]. The optical trapping of large particles is hence limited to relatively simple shapes (e.g., sphere and ellipsoid) or particles whose light scattering properties change slowly with the direction of the trapping beam [7]. No matter in which trapping regime, force calibration or the evaluation of the force-displacement relationship has become a necessary routine in quantitative studies with OTs. Various calibration methods have been proposed and are continuously being improved. As indicated by the aforementioned theoretical simulation shown in Figure 2, the optical force depends linearly on the relative displacement of the particle to the equilibrium position in an optical trap as with a simple spring system, thus it can be characterized by a spring constant. Two common techniques for obtaining the spring constant are passive calibration, where the trapping stiffness ($\kappa_{x}$) is obtained by monitoring the intrinsic movement of the trapped particle within a trap, and active calibration, where an external force is applied to induce a displacement of the particle in an optical trap [76]. Among the passive calibration methods, the power spectrum analysis is regarded as the most reliable one, especially with spherical particles [77]. The power spectrum of Brownian motion of a dielectric bead in an optical trap is monitored by a position detection system (PDS) and modeled with Lorentzian fitting to obtain the corner frequency ($f_{c}$), which is defined as [77]:

(7) $$f_{c}=\frac{\kappa_{x}}{2\pi \gamma }=\frac{\kappa_{x}}{12\pi^{2}\eta \;\cdot \;r},$$

where $\kappa_{x}$ is the trapping stiffness, γ=6πη·r is the particle friction coefficient determined by the trapping medium, η is the medium viscosity coefficient and *r* is the bead radius. The precision of the trapping strength calibrated with this method is limited mainly by the resolution of the PDS.

The typical active calibration is based on the comparison between the trapping force and a known external force, for instance, the Stokes’ dragging force applied by a fluid flow with known velocity ($v_{fluid}$) [2]. The flow can be generated by the controlled movement of the sample stage or by micro-pumping the fluid into a microfluidic chamber. For a small displacement within the trapping range, the trapping force acts against the dragging force described by Stokes’ law [78]:

(8) $$F_{drag}=\gamma \;\cdot \;v_{fluid}=6\pi \eta \;\cdot \;r\;\cdot \;v_{fluid},$$

which tends to push the particle out of the trap. In the equilibrium position, the optical restoring force equals to the dragging force:

(9) $$F_{trap}=\kappa_{x}\;\cdot \;x=F_{drag},$$

where *x* is the displacement of the particle from the trapping center.

To calibrate the optical forces exerted on an arbitrarily (nonspherical) shaped particle (e.g., red blood cell and biological particles) by an arbitrary beam in a poorly known environment, another methodology known as “absolute calibration“ has been established and shown great practical capability in complex biological applications [79]. This method focuses on the deflection of the transmitting beam itself, as illustrated in Figure 1d that a transverse displacement of the particle causes a lateral deflection of the laser beam. The optical force is deduced from the measurement of the calibrated position shift of the “centre of mass“ of the laser beam (i.e., the intensity-weighted average position) by charge-coupled device (CCD) camera or position-sensitive detector (PSD) [80]. In addition to the force-displacement relationship, the calibration of the relationship between the laser power and trapping strength at the equilibrium position in an optical trap is essential in RBC studies to apply controlled trapping strength by adjusting the incident laser power. This can be performed by a method similar to active calibration based on the force equilibrium between the optical trapping force and viscous friction force in a fluid flow environment. With a given trapping power, the velocity of the flow can be slowly increased until the viscous dragging force matches the trapping strength and causes the particle to escape from the trap. The trapping force proportional to the trapping power can then be estimated by the dragging force at the escaping velocity [73].

### 2.4. Thermal and Nonthermal Damage to Biological Objects by Optical Trapping

To create a high gradient profile that is capable of exerting optical force of tens of piconewtons on micrometer-sized particle to achieve 3D noncontact optical trapping, a high power intensity exceeding megawatts (1 MW = $10^{6}$ W) per square centimeter (MW/cm${}^{2}$) at the focused trapping spot will be applied by trapping power of tens to hundreds of milliwatts (mW) [81]. Therefore, in trapping and manipulation of living cells including RBCs, possible thermal damage, as well as the potential impact on the experimental observation induced by the trapping laser have to be considered. Additionally, the temperature drift engendered Brownian motion is one of the main sources of noise that limit the resolution of OTs-based instruments [81]. Concerns and investigations of thermal and nonthermal effects on biological samples have been taken into consideration since the first application of the OTs to biological particles [7,82]. With the help of temperature-sensitive fluorophores, the heating effects of liposomes and Chinese hamster ovary (CHO) cells caused by an infrared (1064 nm) trapping laser (power density: ∼$10^{7}$ W/cm${}^{2}$ and trapping spot size: ∼0.8μm in diameter) have been theoretically modeled and experimentally measured by detecting the fluorescence spectra from the dye-labeled structures [14]. The temperature increase that is linearly proportional to the incident trapping power is found to be 1.45 ± 0.15 ${}^{\circ }C$/100 mW in liposome vesicles, 1.15 ± 0.25 ${}^{\circ }C$/100 mW in CHO cells and less than 1.0 ± 0.30 ${}^{\circ }C$/100 mW in human sperm cells with trapping power up to 400 mW [14,15,83]. Comparable results of temperature rise of about 3.97 ${}^{\circ }C$/100 mW in trapped polystyrene particles in glycerol solution (extinction coefficient of ∼20 m${}^{-1}$) and of about 0.79 ${}^{\circ }C$/100 mW in trapped silica beads in water solution (extinction coefficient of ∼10 m${}^{-1}$) have been obtained by monitoring the Brownian motion spectra of the laser-confined particles [81]. Suggested by some studies, the sample heating within the focus can be roughly estimated by Fourier’s law of heat conduction [84,85]. Detailed theoretical models for calculating the temperature changes of the trapped absorbing particles in OTs have been established under the approximation that the trapped object is an infinite medium with homogeneous thermal characteristics and with the consideration of particle absorption [14,81,86]. Other potential nonthermal cell damages caused by linear optical excitation, nonlinear photonic effects and reactive oxygen species/singlet oxygen as a consequence of high-intensity near-infrared laser radiation need to be considered when trapping biological objects [16,87]. In optical trapping of human RBCs, except for the undesirable temperature rise, studies have revealed the OTs-induced deoxygenation of the trapping site on the RBC surface that is proportional to the trapping power [88] and have observed cell membrane ionization, inactivation and ejection from the trap by the thermally produced (radiometric) force induced by high power infrared laser trapping [89,90,91]. The observed RBC membrane rupture under high laser power (>500 mW) has been proven to be caused by the membrane electro-permeabilization and the resulting strong temperature gradient across the cell membrane induced by shape changes, rather than the total temperature rise (1.4 ${}^{\circ }C$/100 mW) around the cell [89]. Importantly, such biological damage is not restricted to the trapped cell, the sudden and rapid collapse of the trapped RBC will cause shock waves that will further exert a mechanical damaging impact on adjacent untrapped cells within a circular “damage zone“, the size of which is expanded with the increase of the trapping power [89].

In general, a temperature rise of less than 1.0 °C is expected when the trapping power is no more than 100 mW in an infrared (1064 nm) optical trap. Typically, infrared laser trapping results in reversible sample heating of several degrees centigrade in living cells without causing thermal damage. The upper limit of temperature increase by complete laser absorption can be estimated using the Stefan–Boltzmann law: $I=\sigma \;\cdot \;T^{4}$, where σ is the Stefan–Boltzmann constant equals to $5.67\times 10^{-8}$ W/(m${}^{2}$K${}^{4}$) [92]. It is important to note that the time it takes for the temperature to rise and fall depends on the heat conduction rate away from the locality of heating. In the stable trapping stage, a quasi-thermal-equilibrium condition is initiated, hence the sample temperature is sustained during the trapping process [14]. Practically, the trapping wavelength and power should be chosen to minimize thermal absorption by both the trapped cells and the surrounding medium to avoid potential damage. Typically, the near-infrared (750–1400 nm) wavelengths (e.g., Nd:YAG laser at 1064 nm, Nd:YLF laser at 1047/1053 nm and tunable Ti:Sapphire laser at 700–1100 nm) are selected in blood cells trapping and analyzing in accordance with the transparent window of the blood absorption spectrum [18].

## 3. Studies of Human RBCs by OTs

The human circulatory system that permits blood to circulate and transport nutrients, gasses, hormones and waste products to and from the cells in the body is known to play a vital role in supporting and regulating organ functions as well as indicating overall health and disease conditions [93]. The micro-rheological properties of RBCs, as one of the main blood components, significantly affect blood microcirculation and hence have a direct bearing on the circulatory functioning [84]. Traditional methods for blood sample analysis through optical transmission or reflection properties of cell suspensions are based on an average response of a large number of cells [94] and are unable to reveal detailed information about single-cell properties and individual cell-cell interaction mechanisms. Currently, single-cell level methods, such as micropipette aspiration technique (MAT), scanning electron microscopy (SEM) and atomic force microscopy (AFM), have been applied to RBC studies and have contributed a lot to the current understanding of the special characteristics of RBCs and blood rheology [95]. To date, many aspects of RBCs, including membrane elasticity [96], disaggregation dynamics [73], shape and electrical charge of the membrane surface [97], as well as the special characteristics under different physiological and pathological conditions [98,99,100,101] have been investigated with various OTs systems. The main research methods and results in RBC studies achieved by OTs are reviewed to provide inspiration for novel experimental design of OTs measurement systems and to spur potential new applications of OTs in blood studies.

### 3.1. RBC Preparation and Trapping

**Figure 5:**
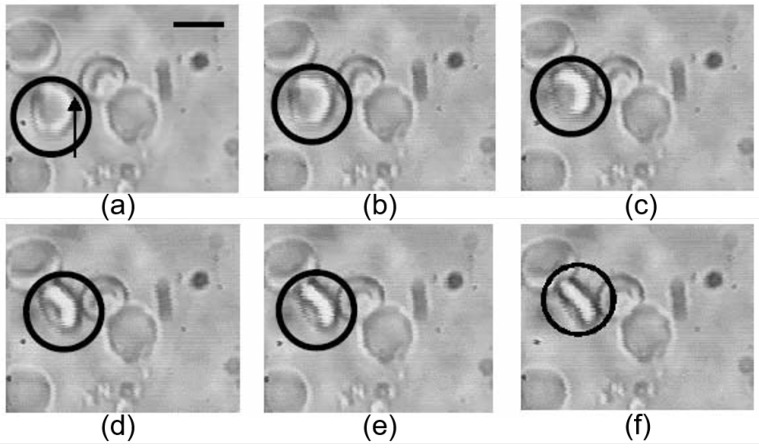
Optical trapping of a healthy mature RBC (circled) from the horizontal position (**a**) to the vertical direction (**f**) by an infrared trapping beam of 85 mW (location is shown by arrow) [106]. From (**a**)–(**f**), the time interval between each of the two photos is 40 ms.

The experimental samples used for general RBC measurements with OTs are erythrocytes suspensions with controlled concentrations. Normal circulating human RBCs can be obtained by washing the fingertip-prick blood drops in phosphate buffer saline [102] and for evaluation of the maturing RBCs during different development stages in erythropoiesis; the immature RBCs can be obtained by aspirating bone marrow cells from the iliac crest [103]. The cells are suspended in designed solutions for different measurement purposes, such as plasma or model solutions containing different types and concentrations of fibrous biomolecules, neutral polymers or nanoparticles. Hypertonic buffer can be used to get swollen spherical or nearly spherical RBCs when needed [104]. To perform experiments with indirect RBC trapping and manipulation, silica or polystyrene microbeads are incubated with RBC suspensions to adhere to the RBC membrane and act as “handles“ [105]. The sample chamber can be made of a microscope slide and coverslip that are connected through a double-sided sticking thin film (50–100 μm). When necessary, bovine serum albumin (BSA) can be used to coat the slides and/or coverslips to prevent RBCs or microbeads from adhering to the chamber walls. The RBCs are normally at rest on the surface of the microscope slide, and once the laser is turned on, the selected cells can be trapped and lifted from the bottom of the sample chamber. The trapping process of a healthy RBC by an infrared trapping beam in buffer solution is shown in Figure 5 [106]. It can be seen that due to its biconcave shape, the RBC turns to the side after being stably trapped—this phenomenon is also referred to as RBC getting folded into an optical trap [107]. The trapping dynamics of healthy RBCs suspended in buffer solutions with varying osmolarity have been studied, and the time course required to achieve stable optical trapping of a single RBC was discovered to be dependent on both the trapping power and the suspension osmolarity [106].

### 3.2. Evaluation of RBCs Developing and Functioning

In terms of erythropoiesis, the RBCs experience complete changes in cell composition and membrane mechanical properties during the journey of RBC production from the immature pronormoblasts to the mature biconcave discocytes [108]. Studies have shown that measurements of membrane mechanical attributes of the developing RBCs indicate the structural and functional maturation of the membrane skeleton [103]. Moreover, the erythropoiesis system is involved in and notably reactive to erythroid stress and a series of hematological conditions, including hemorrhage, hemolysis, hypoxia and anemia and will be inhibited under certain pathological conditions [109]. For instance, it has been assumed that the accelerated apoptosis of the premature erythroid cells caused by the deposition of excess α-globin chains and the restricted erythroid cell differentiation are the causes of the ineffective erythropoiesis in β-thalassemic syndromes [110,111]. The OTs-based techniques, including conventional infrared OTs, polarized OTs and hybrid Raman tweezers have demonstrated unique sensitivity and advantages in screening and diagnosing a variety of diseases and thus possess special potential in evaluating the efficiency and outcome of erythropoiesis, as well as in discovering the origin of altered erythropoiesis under pathological conditions.

#### 3.2.1. Applications of Raman Tweezers

**Figure 6:**
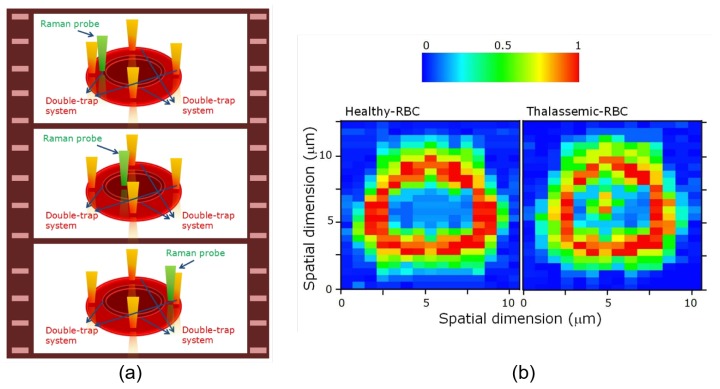
(**a**) Illustration of the experimental procedure of Raman imaging of the equatorial plane of a RBC hold by four infrared (1064 nm) optical traps by a Raman excitation probe (532 nm) with scanning step-size of 0.5 μm. (**b**) Reconstructed hemoglobin distribution within healthy (left) and thalassemic (right) RBCs in cell equatorial plane. Normal hemoglobin distribution presents classical ring-shape, whereas the possibility to detect hemoglobin within the central area increases in diseased RBC [113].

OTs are highly compatible with other measurement modalities, including Raman spectroscopy, fluorescence microscopy, absorption and photoluminescence spectroscopy [112]. The Raman spectrum based on the inelastic light scattering determined by molecular vibrations is capable of revealing the intrinsic molecular information, including the chemical constitution and structural conformation of macromolecules [113]. However, to avoid random displacing of cells from the confocal excitation volume, cell immobilization is required either physically or chemically. By combining OTs with Raman spectroscope, novel Raman tweezers have enabled the inelastic light scattering measurement from noninvasively fixed target cells one by one with great operation freedom and have shown wide potential in various biomedical and clinical applications. Specifically, diagnostic models for epithelial cancer [112], thalassemia (a hereditary hemolytic disease) [113,114] and type II diabetes [115] based on identification and discrimination of single cells of pathological samples by Raman tweezers have been developed. In Raman tweezers, the trapping and Raman excitation wavelengths can be selected separately according to sample properties and excitation requirements [113], whereas in some applications, the trapping laser was utilized simultaneously as Raman excitation beam [115]. With Raman tweezers, the altered protein constitution and hemoglobin oxygenation in abnormal or diseased RBCs can be detected by the significant variations in Raman spectra of noninvasively optically trapped RBCs without exogenous markers, enabling Raman tweezers to be a promising, convenient and label-free tool for accurate monitoring of hemoglobin-related blood disorders, including type II diabetes and α- and β-thalassemia [113,114,115]. As illustrated by Figure 6a, by scanning the equatorial plane of an RBC controlled by four optical traps through a Raman excitation probe (532 nm), the biomolecular characterization of single RBCs (i.e., hemoglobin distribution mapping) through recorded Raman spectra can be realized, and the healthy and thalassemic-RBCs can be noninvasively differentiated [113]. Compared to conventional thalassemia screening methods including Fourier-transform infrared (FT-IR) spectroscopy that require strict laboratory preparations and involve unwanted hemoglobin structural changes, Raman tweezers not only provide a simple, noninvasive and easy-to-implement alternative for thalassemia diagnosis, but also help with the investigation of the origin of the ineffective erythropoiesis in β-thalassemia. In addition to cell immobilization, OTs can apply adjustable optical forces on the trapped cells to study the degree of deoxygenation induced by chemical or mechanical pressure (known as mechanochemical phenomenon) from the variations in the spectral intensity of several oxygenation-specific Raman peaks [116,117,118]. Other investigations including ABO blood typing [119] and malaria-infected blood sample characterization [120] have been performed with Raman tweezers. Raman spectroscopy has also been demonstrated efficient in discerning neoplastic cells from normal hematopoietic cells, making hybrid Raman tweezers a potentially powerful and sensitive tool for noninvasive single-cell cancer detection [121]. Indubitably, Raman tweezers, with the capability to differentiate healthy and abnormal RBCs accurately by detecting small changes in Raman spectra induced by mechanical, electrical, chemical and/or structural alterations in single RBCs, are highly promising for the future precise and noninvasive clinical diagnosis and for testing therapeutic responses to blood-targeted drugs.

#### 3.2.2. Applications of Conventional and Polarized OTs

**Figure 7:**
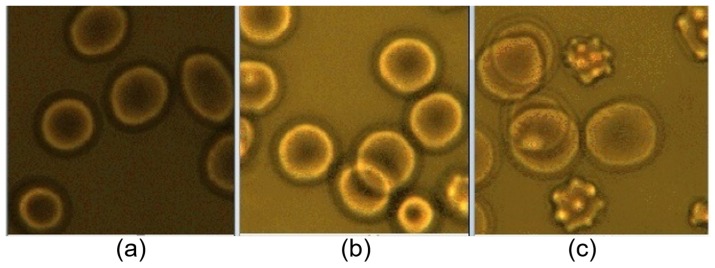
Blood samples diluted in fetal bovine serum for blood transfusion treatment evaluation with OTs: (**a**) normal fresh sample, (**b**) normal stored sample and (**c**) transfused SCA sample [124].

With conventional OTs-based methods, diagnostic and therapeutic models of certain diseases, including sickle cell disorders, have been established based on the direct evaluation of RBC mechanical and physical properties [90,122,123,124]. The efficacy of blood transfusion treatment in sickle cell anemia (SCA) has been evaluated by analyzing the mechanical response of transfused RBCs measured by conventional OTs [124]. As demonstrated by the experimental samples shown in Figure 7, the SCA RBC sample possesses obvious morphological changes compared with normal blood. The RBC elasticity has been obtained by dragging optically trapped RBCs in a viscous fluid environment and measuring cells’ elastic response (elongation) as a function of dragging velocity. The healthy and haemoglobin S (HbS) mutation samples (sickle cell disease) can be easily differentiated according to their differed flexibility, which suggests the deformability as one of the main contributors to clinical responses of HbS mutations [122]. Furthermore, the effect and efficiency of drug treatments, such as hydroxyurea (HU) and atorvastatin (one of the most common statin drugs), on the mechanical properties of RBCs, or in a broader context, on the biomechanics of human cells, have been tested with OTs-based deformability measurements [122,125]. Recently in 2019, the micromechanical responses of RBCs under different oxidative stresses, which is one of the most influential causes of Parkinson’s disease and can cause direct oxidative damage on RBCs, have been studied with high sensitivity and accuracy by OTs, indicating RBC elasticity as a potentially significant indicator for early Parkinson’s disease monitoring [126].

**Figure 8:**
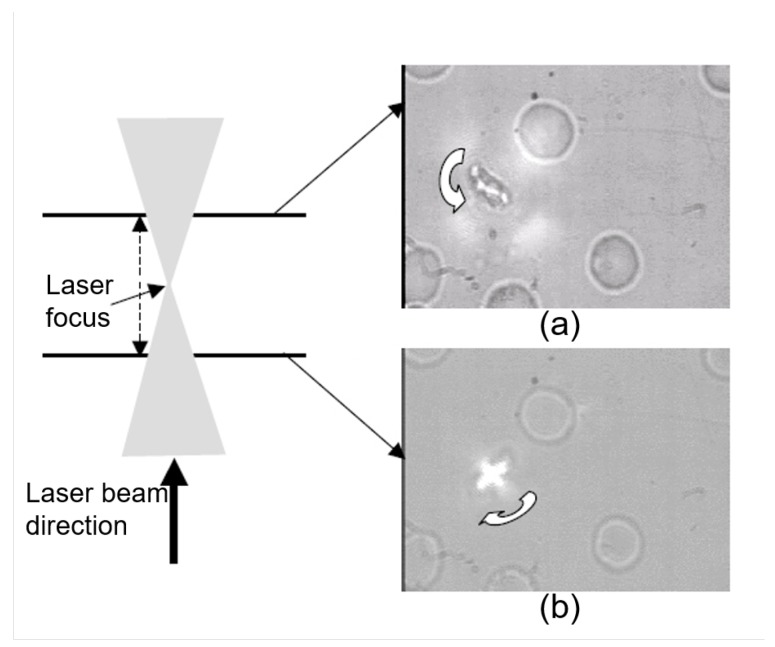
Experimental observation of the rotation of Plasmodium-infected RBCs in linearly polarized trapping beam [128]. (**a**) Anti-clockwise rotation is observed when the iRBC is below the beam focus and (**b**) clockwise rotation with the same rotation speed and the unchanged laser power is recorded when the iRBC is before the focus along the laser propagation direction.

Other studies have focused on the OTs measurement of RBC interaction dynamics and successfully found the relationship between the altered intercellular interaction forces and various pathological conditions including systemic lupus erythematosus (SLE). The measured aggregation force between RBCs in pair rouleaux was found to be larger in SLE patients than in normal samples, and the aggregation speed for SLE RBCs was nearly two times higher than that for normal RBCs [98]. A recent study from our group has examined the influence of different inorganic and polymeric nanoparticles on the RBC interaction dynamics. Among all of the tested nanoparticles, only the nanodiamonds induced an increase in the size of RBC aggregates and the aggregation force [127]. Such tests have practical significance for ensuring the safe use of novel nanoparticles in biomedical applications, including nanomedicine and photodynamic therapy (PDT). Intriguingly, special behavior of cell rotation of Plasmodium-infected (malaria) red blood cells (iRBCs) in the polarized trapping light field as shown in Figure 8 has been reported [128], whereas healthy RBCs only show alignment along the polarization direction of the trapping light. The rotation speed (19–300 rpm) is related to the infection degree and trapping power. The altered transmembrane mobility for different ionic species (Na${}^{+}$, Ca${}^{2+}$ and K${}^{+}$) is considered responsible for enhanced anisotropy of the polarizability tensor of the iRBCs. The proposed polarized OTs-based method provides a sensitive and easy-to-implement tool to distinguish iRBCs from normal cells. In addition to the disease monitoring methods based on the measurement of basic cell properties, a novel optical diagnostic mechanism based on the speed of membrane damage and collapse under high infrared (1064 nm) trapping power (>280 mW) has been proposed and successfully applied to diabetic detection [89]. Recently, an easy way to detect malaria-infected RBCs through the prolonged time course required to achieve stable trapping and the increased Brownian motion of infected cells inside a trap has been proposed [107]. The altered trapping behavior of malaria-infected RBCs is assumed to be due to the increased membrane rigidity influenced by the releasing of a malaria-specific substance in the bloodstream regardless of whether they were hosting parasite or not and thus the method can be applied to early-stage malaria diagnosis. The possible inducers of the risen rigidity in malaria-infected RBCs were examined with the same method [129].

### 3.3. Evaluation of RBC Membrane Deformation

Immature RBCs experience dramatic changes in shape, cellular composition, and membrane mechanical and physical properties throughout the erythropoietic process [103]. The quantitative evaluation of the stiffness of the nucleated and enucleated RBCs during maturation enables the direct evaluation of the development of membrane skeleton [108]. Mature human red blood cells (RBCs) are unique mammalian cells in biconcave shape (typically 6–8 μm in diameter and 2 μm in thickness) that contain no nucleus or subcellular metabolic structures [130]. They can be regarded as a concentrated hemoglobin solution enveloped by a highly flexible membrane and are responsible for transporting gasses (e.g., oxygen and carbon dioxide), nutrients and wastes through circulating vessels of various sizes. The RBCs are highly deformable biomaterial that can undergo large deformations under external stresses, which is crucial for an appropriate vascular function and adequate organ perfusion as the red cells have to pass through capillaries narrower than the size of a resting RBC [131]. With OTs that are capable of accurate single-cell manipulation, the RBC deformation mechanics have been studied under various precisely controlled mechanical and environmental states. The elastic properties of the RBCs membrane including the area expansion, elastic shear moduli and the bending stiffness, have been studied from the mechanical responses of the RBC membrane to the applied optical forces, as well as from the restoration process following the release of the cell from the optical stretching [96,97]. Particularly, regarding the RBCs ontogeny, RBCs undergo different cellular morphologies during the different stages of erythropoiesis. The membrane flexibility of the stress-free spheroidal shaped premature RBCs and their motion in shear flow can be analyzed by OTs experiments [132]. The optical forces can be applied directly to several points on the RBC membrane [133,134] or through several silica beads bound to the RBC membrane to reduce possible heating of the trapped cells [104,105]. Using microbeads as handles further provides possibilities of optimal selection of the number, size and distribution of the beads, supplying supplementary flexibility and compatibility for utilizing OTs in cell mechanical characterization.

**Figure 9:**
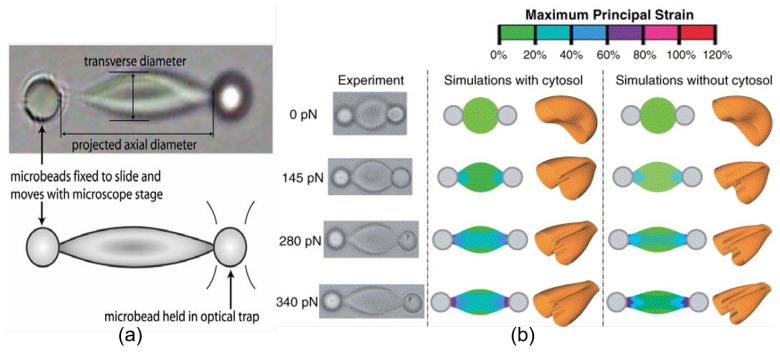
(**a**) Illustration of OTs-induced RBC deformation through silica microbeads (4.12 μm in diameter) attached to cell surface [135]. The right bead is optically trapped while the left bead is fixed on the microscope slide and moved away from the right bead by displacing the glass slide to realize cell stretching. The cell deformation is denoted by the changes in both RBC transverse and axial diameter. (**b**) The experimental demonstration of the RBC deformation under different optical stretching forces up to 340 pN and the corresponding theoretical strain distribution within deformed RBC simulations with/without the existence of cytosol [96].

The study of RBC deformability with OTs was pioneered in the 20th century and a two-channel OTs system based on the rapid commuting of the trapping beam between two points was used to measure the membrane elasticity [104]. By stretching a single RBC from two diametrical positions, the elastic shear modulus of 2.5 ± 0.4 μN/m was deduced from the slope of the linear relationship between the membrane equatorial deformation and the applied force. Figure 9a illustrates the typical cell stretching applied by OTs through silica beads attached to the red cell membrane [135]. With the same beam shifting method, an OTs system with up to four trapping channels was used for the direct measurement of the deformability of the two-dimensional spectrin network underlying the cell membrane by dissolving the lipid bilayer after the successful trapping of RBCs through silica beads that were bound to the spectrin skeleton [105]. With well-controlled position manipulation of three optical traps, different deformation conditions, including the pure area expansion, pure shear and combined shear and area expansion were generated and the measured area expansion modulus of 4.8 ± 2.7 μN/m and shear modulus of 2.4 ± 0.7 μN/m in low hypotonic buffer were very consistent with theoretical predictions [105]. Later, a refined and extended OTs-based system for investigating the large deformation properties of single RBCs was proposed. The optical stretching force up to 600 pN was applied to trapped cells through silica microbeads to study the effects of different cell-specific (e.g., interior volume and diameter) and environmental factors on RBC large-scale deformation systematically and parametrically [96,136]. Experimental observations of optical stretching of single RBCs and the corresponding simulation results of RBC deformation under different external tension obtained by a three-dimensional finite element model are illustrated in Figure 9b [96]. By evaluating the RBC deformability by OTs, RBC aging during in vitro storage for blood transfusion purposes has been evaluated quantitatively in recent years. It is discovered that the elastic shear modulus of the RBC membrane measured by the OTs-based stretching method increased from 2.5 μN/m to 13 μN/m over 21-day storage at 4 °C after donation [137]. The phenomenon of RBC membrane stiffening has been further confirmed and investigated in detail for 15-day storage by evaluating the elongation of the optically trapped RBCs under various dragging velocity against the blood serum [138]. With a microfluidic platform that consists of a constriction channel capable of inducing the deformation shear stress to RBCs, the RBC deformability deterioration was studied for up to 42 days of storage [139]. In summary, OTs have been proved an accurate and reliable method to study RBC deformation in both the small strain linear elasticity regime (typically trapping force F < 15 pN) and the large-scale deformation evaluations. The response of the cell membrane as a whole and the respective roles of under-membrane structures in RBC deformation can be studied separately and precisely with OTs at a single-cell-level. Consequently, the OTs-based methods provide powerful experimental platforms for studying the elastic and viscoelastic deformation of living cells under different mechanical, chemical and biological environmental states with great flexibility.

### 3.4. Evaluation of Dynamic Cell-Cell Interaction between RBCs

**Figure 10:**
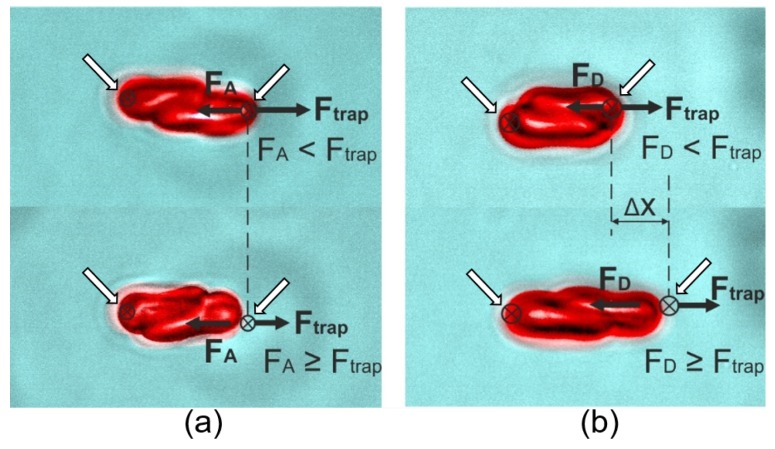
Illustration of measurements of RBC mutual interaction forces in RBC aggregates containing two cells [102]. (**a**) The aggregate is slightly stretched by two opposite end-points and the RBC aggregation force is measured by slowly decreasing the trapping strength until the optical force matches with the intercellular force and the cells escape from the traps. (**b**) The optical pulling force required to separate two RBCs is measured by slowly displacing the right trap away from the fixed left trap to destruct the original contact between the two cells until the cells escape from the traps. The white arrows indicate the trapping points (black crosses inside black circles).

The RBCs have a well-known intrinsic tendency to form two/three-dimensional binding structures when the mutual attractive force (e.g., hydrophobic and hydrogen bonds, van der Waals forces) is greater than repulsive interactions (a consequence of membrane charges) [140]. RBC aggregation depends on several factors including cell-specific properties and characteristics of the suspending environment [141]. The properties of RBC aggregation including degree, speed and size of formed aggregates are important hemorheological determinants that have a direct bearing on the microcirculation and are critical for hemostasis when wounds appear outside or inside the human body [142]. It is important to understand the RBCs interaction mechanism and the contribution of different factors to RBC disaggregation to develop better monitoring and therapy of blood microcirculation in the future. Currently, there are two coexistent yet mutually opposed theoretical hypotheses, the “depletion layer“ model and the “cross-bridging“ model to describe the mechanisms involved in RBC spontaneous aggregation and enforced disaggregation [143]. The “depletion layer“ model attributes the RBC clumping to the osmotic pressure originating from a layer of low macromolecule concentration near the interaction surface and considers the interaction force to be proportional to the interaction area and uniformly distributed throughout the contacting area [144]. On the other hand, the “cross-bridging“ model indicates that two adjacent RBCs are connected by the “bridges“ formed by the macromolecules attached to the membrane of both cells, thus the strength of the interaction is associated with both the type and concentration of macromolecules in the surrounding environment [145]. In OTs-based RBC interaction measurements, the aggregation force is measured as the minimum trapping force to stop RBCs from clumping as illustrated in Figure 10a, and the disaggregation force is measured as the optical pulling force applied to break RBC bonds and decrease the contact area between the two cells as shown in Figure 10b [102]. In the prototypical procedure of RBC aggregation force measurement, linear RBC aggregate consisting of two or several cells is held and slightly stretched from two opposite end-points, and the trapping strength is slowly decreased while the interaction area remains constant. The aggregation force is estimated as the optical trapping force when the interaction force exceeds the trapping force and the corresponding end of the aggregate escapes from the trap. In disaggregation force measurement, with a given trapping power, one trap is slowing moving away from the other trap until the interaction force (disaggregation force) overcomes the trapping force and draws the end of the aggregate out of the trap.

With the double-beam OTs system as illustrated in Figure 4 and the above-stated methods, our group recently revealed the influence of cell interaction time (up to 300 s) on RBC disaggregation dynamics [73,102]. The results showed that the RBC disaggregation force increases with initial interaction time between two RBCs, whereas the aggregation force is independent to the cell contact time. The main difference between RBC aggregation and disaggregation forces is that the aggregation force is typically much weaker than the disaggregation force and is uniformly distributed throughout the interaction area [146], whereas the disaggregation force is reciprocally related to the relative interaction area [94]. The interaction history (previous overlapping area) between two RBCs has also been manifested to have an effect on the optical force needed to dissociate RBC aggregates [146]. The interaction force between RBCs in linear aggregates containing more than two cells has also been measured with the same method [84]. The roles of various cell-specific and environmental factors in regulating RBC mutual interaction have been systematically studied and the aggregation behavior has been analyzed in a wide variety of solutions with agglutination potentiators, including dextran, low ionic strength solutions (LISS), enzymes (bromelain and papain), RBC antibodies and normal serum [140]. The influence of different protein solutions (e.g., fibrinogen and albumin) with different concentrations on RBC interaction dynamics and the biphasic role of the concentration of dextran (150 kDa) in inducing RBC aggregation have been revealed [94,147]. In comparative studies of OTs and other cell measurement techniques, the RBC aggregation shear stress measured with OTs is very consistent with the RheoScan aggregometer analysis based on light scattering properties of the whole blood sample, whereas the disaggregation shear stress measured in individual RBC doublets is significantly higher than that measured by aggregometer [148]. Moreover, the comparison of the RBC aggregation shear stress measured with OTs and other shearing geometries at different temperature conditions (in a range of 22–38 °C has proven that OTs have great measurement stability and can eliminate the interference of ambient temperature to the measurement results [149]. In addition to interaction evaluation in individual cell-pairs, the intercellular attraction applied on a single RBC by a large aggregated RBC group has been measured by OTs as the minimum optical trapping force to stop an RBC from coagulating to the group [142].

**Figure 11:**
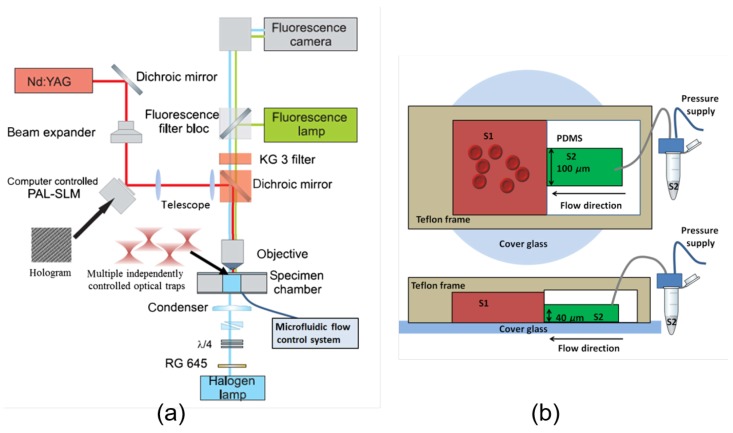
HOTs integrated with a microfluidic platform for assessing RBC aggregation mechanisms in changing solutions [78]. (**a**) Schematic diagram of the HOTs with four independent trapping channels. (**b**) Top view (top) and side view (below) of the microfluidic system (not in scale). RBC aggregates formed in solution 1 (S1) are trapped and moved to solution 2 (S2) for aggregation force measurements.

**Figure 12:**
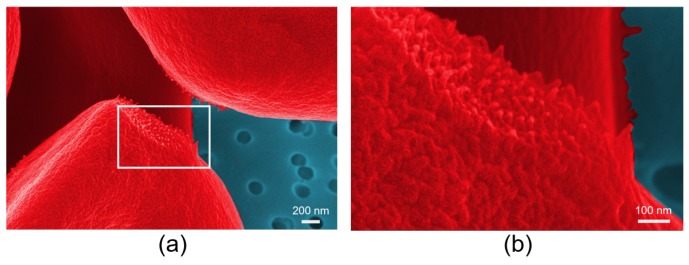
(**a**) RBC visualization by SEM reveals discrete cilia on the cell surface. (**b**) Enlarged detail of the inner part of the white rectangle in (**a**) indicates the cilia density as 1/3600 nm${}^{2}$ [151].

The development of advanced trapping approaches has encouraged the design of novel experimental methodologies that integrate the OTs with microfluidic platforms or other microscopic techniques for in-depth investigation of RBC interaction dynamics in the well-controlled single-cell environment. Based on HOTs and the fact that a microfluidic platform consists of two connected chambers as shown in Figure 11, the RBC aggregation force can be ingeniously measured in changing solutions (plasma, phosphate buffer saline, protein solutions of fibrinogen and/or albumin) with designed order to explore the aggregation mechanism [78]. The interesting observation that RBC adhesion is strongly dependent on the initial aggregate-forming solution provides new evidence of the involvement of the “cross-bridging“ mechanism in the RBC aggregation process. The role of macromolecule absorption in altering RBC mechanical properties and hence in influencing aggregation dynamics has been studied in detail with the same HOTs integrated with a fluorescence microscopy [150]. Recent works from our group have visualized the membrane morphologies of RBCs that adhered to each other by scanning electron microscopy (SEM) as shown in Figure 12 and evaluated the adhering strength in dextran solutions of different molecular weights (70 kDa, 150 kDa and 500 kDa) by conventional dual-beam OTs [151,152]. The SEM visualization of RBCs in mutual interactions has enabled the direct observation of the cilia on the cell surface, and the calculated cilia density is in good agreement with the deduced cross-bridges density in the “cross-bridging“ model. Indeed, the OTs hold superior advantages in investigating RBC interaction dynamics and exploring the causes of abnormal agglutination in various diseases. The development of advanced OTs-based measurement techniques and their great application potentiality in exploring cell interactions will continuously improve the understanding of blood rheology and immunohematology and further contribute to the development of future microcirculation monitoring and treatment solutions.

## 4. Evaluation of RBCs Electrical Properties

**Figure 13:**
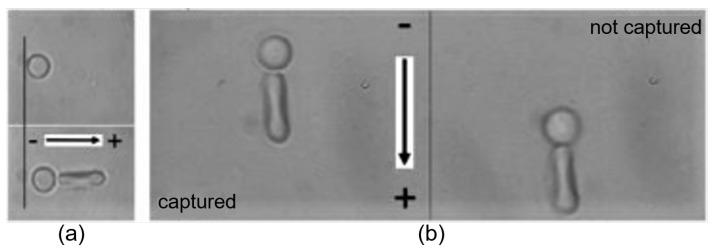
Measurement of RBC surface potential by (**a**) matching optical trapping force with electric pulling force and by (**b**) monitoring the moving velocity of a RBC released from an optical trap in an electric field [97]. Small sphere indicates the silica bead attached to the RBC (seen as rod in shape).

Attributes to the nature of negatively charged RBC surface as a result of the existence of glycolipids in the fluid lipid bilayer of the cell membrane, the zeta potential between RBCs induced by the surrounding cloud of oppositely charged ions (known as the double layer) prevents cells from spontaneous aggregation and formation of undesired clots in bloodstream in normal conditions. The RBC electrical property measurement is typically based on the movement of negatively charged RBCs in an external electric field applied to RBC suspensions in an electrolytic solution. The constant terminal moving velocity that is dependent on the applied voltage can be used to calculate the zeta potential of individual RBC. OTs can be utilized to select, release and recapture a single RBC in such measurements and to directly measure the electric force applied on a trapped cell by matching the optical trapping force with the electric dragging force, as shown in Figure 13 [97]. With direct electric force measurement, the zeta potential and the thickness of the double layer of individual RBCs have been successfully estimated [97]. With the terminal moving velocity measurement method, a decrease of 42% in RBC zeta potential after 15-day storage was observed [138]. In particular, some studies have reported the phenomenon of RBC ejection from an optical trap as a mutual result of trapping force, viscous friction force that is applied by the suspending medium and the laser-induced electrostatic force due to membrane ionization under high infrared (1064 nm) trapping power [90,91]. According to the RBC ionization energy, the time required for ionization and the number of charges formed on the cell surface measured with OTs, RBCs containing two different types of hemoglobin are differentiated as a result of the fact that different kinds of hemoglobin carry a different amount of charges [90]. The obtained results have enabled a better understanding of the relation between the cell charge and the electrolytic environment, and the proposed methodology with OTs provides an effective tool for comprehensive studies of the electrical properties of both RBCs and other types of biological samples.

## 5. Cell Trapping and Manipulation In Vivo

**Figure 14:**
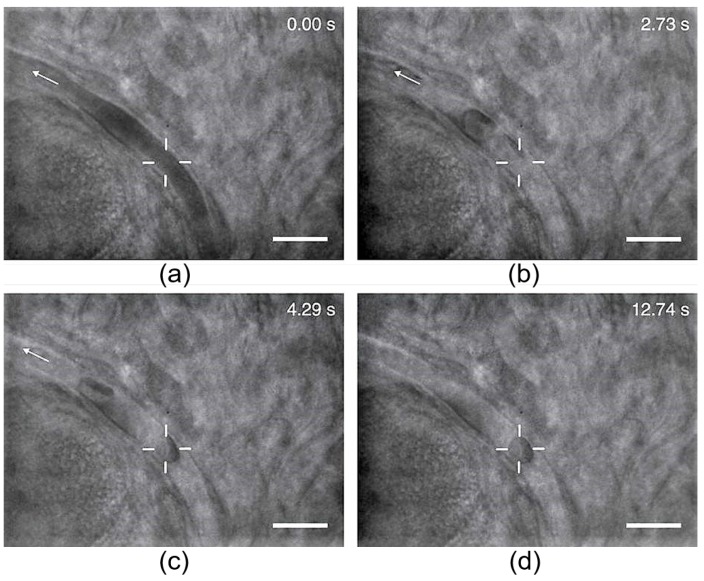
In vivo RBC trapping in a small capillary of a mouse ear [153]. (**a**) Single red blood cells (RBCs) are indistinguishable in the blood flow. (**b**) After several seconds of applying the OTs, the blood flow slows down and individual RBCs can be seen. (**c**) Successful trapping of a single RBC when the trapping force overcomes the flow dragging. (**d**) Stable trapping of the cell for over 10 s without influencing the normal flow of untrapped RBCs. White “+“ indicates the trapping position and the arrows show the blood flow direction with scale bars of 10 μm.

The majority of OTs-based experiments are performed in vitro. However, the results obtained in vitro cannot accurately and truly reflect the comprehensive biological activities in vivo. To perform in vivo trapping of living cells, several obvious difficulties need to be thoroughly considered. First, the in vivo optical force calibration is more complex than in vitro due to the variations of the trapping environment at different tissue locations. Secondly, biological tissues have strong absorption and scattering properties, which makes it hard to deliver sufficient trapping intensity deep into the tissue without causing thermal or photo-damage. The aberration in focusing through beam propagation in biological tissue further adds the complexity of in vivo trapping. Noninvasive trapping and manipulation of RBCs in vivo with infrared OTs was reported for the first time in 2013 as illustrated in Figure 14 [153]. The RBCs within subdermal blood vessels in living mice were successfully trapped and displaced longitudinally in the blood vessel by about 6 μm, and the optical trapping stiffness was roughly calibrated in mice capillary. The laser power of about 168 mW at the trapping spot was used to trap a single RBC in small capillary (about 5 μm in diameter) at about 40 μm beneath the skin surface of a mouse ear. A slightly higher power was used for RBC trapping in large vessels (about 16 μm in diameter) at about the same depth (45 μm) to adapt to the fast blood flow. As observed in experiments, artificial blood clots that are capable of blocking the capillary can be induced by optical trapping of a single or several RBCs. The artificially formed blood clots can be subsequently dissociated and removed by the same OTs to recover the blood flow in the blocked vessel, indicating the potentially useful application of OTs to fast and direct treatment of thrombosis in superficial blood vessels. However, optical trapping at deep depth over 60μm under skin surface was hard to achieve due to the strong absorption of the trapping power by the biological tissue.

In the last decade, the in vivo manipulations of living cells by OTs have been rapidly developing [154]. Recently in 2018, the in vivo RBC trapping in the specifically chosen vessels in pigment poor areas within living zebrafish embryos without causing biological damage or morphologic alteration has been reported [155]. Transient trapping of a single RBC was achieved inside the dorsal aorta where the blood flow was fast. The permanent holding of RBCs while not affecting the normal passing of other cells was performed in the posterior cardinal vein, where the blood flow velocity was slow. OTs-based in vivo trapping has been proven able to redirect RBCs into the unperfused capillaries to repair blood flow. Furthermore, recent studies have demonstrated OTs as unsurprisingly useful tools in guiding the in vivo cell transplantation. The hematopoietic stem cells (HSCs) in the bone marrow (BM) have the ability to engender the entire hematopoietic system and give rise to all blood cells. The functional characterization of HSCs, as well as the understanding of the interaction between HSCs and the BM environment, are of great scientific and clinical importance as they have made it possible for the manipulation of hematopoietic regulators in manners that have made far-reaching changes in the therapy of blood diseases and in stem cell transplantation [156]. With the optical platform integrating the conventional OTs (800 nm) with a multiphoton microscope and a laser microsurgery unit, the image-guided direct single hematopoietic stem cell transplantation into the bone marrow of live mice has been realized, which enables the controlled in vivo investigation of the normal and malignant stem cells [157].

## 6. Conclusions and Prospects

In conclusion, the development of OTs-based experimental methodologies has advanced the investigation of RBC membrane mechanics, intercellular interactions and cellular constituents, which has further promoted the understanding of their roles in regulating cell growth, development, and apoptosis, as well as in influencing various pathological and physiological conditions [158]. Our understanding of the erythropoiesis or “RBC formation“ has been dramatically boosted with the improvement of advanced scientific methodologies and experimental innovations [159]. From the definite advantages and abundant achievements of OTs in the study of the characteristics of RBCs summarized in this review, it can be predicted that the OTs-based methods will have great application prospects in the future exploration of the mechanisms and origins of erythropoiesis. Other promising applications of OTs in erythropoiesis include developing the stem cell-based therapy, building the in vitro blood cell development systems and aiding the transplantation of exogenous RBC factories into the bone marrow to replace the impaired endogenous to secure RBC supply. As with any tool, there are certain drawbacks and shortcomings of OTs technique in biological research. The trapping wavelength and strength are restricted by the potential thermal and nonthermal damage caused by the trapping light [16]. The difficulties of in vivo applications of OTs arise from the limited penetration depth of the trapping laser in biological tissues [153]. Besides, as a single-cell manipulation method, OTs-based measurements are facing difficulties in collecting enough data for statistical analysis [102]. Nevertheless, such defects can be overcome or minimized by subtle experimental designs. For instance, in a study of RBC deformability, the application of a moving-trajectory controllable dual-axis stage makes it possible to apply tensile deformation to up to 450 RBCs in each experiment with a single-beam OTs setup [160]. In general, the future prospects of the development of OTs-based methodologies in blood cell studies can be discussed from two aspects. On the one hand, novel OTs-based systems in combination with other measurement modalities (e.g., spectroscopic and microscopic techniques) and platforms (e.g., microfluidic and lab-on-chip devices) for better performance and visualization, especially for in vivo studies, will be established. On the other hand, convenient and systemic OTs-based disease screening and diagnostic models will be developed.
